# Zn^2+^-induced electronic polarization and local reactivity in melanin-like dopamine oligomers: a DFT study

**DOI:** 10.1007/s00894-026-06901-6

**Published:** 2026-08-20

**Authors:** João Pedro Dionizio, Henrique Rodrigues Souza-Silva, Jean Valdir Uchôa Teixeira, Orisson Ponce Gomes, Didier Bégué, Paulo Noronha Lisboa-Filho, Augusto Batagin-Neto

**Affiliations:** 1 POSMAT, School of Sciences, São Paulo State University (UNESP), Bauru/SP, 17033-360 Brazil; 2 E2S UPPA, CNRS, Institut des Sciences Analytiques et Physico-chimie pour l’environnement et les matériaux (IPREM), Université de Pau et des Pays de l’Adour, 64000 Pau, France; 3 Department of Physics and Meteorology, School of Sciences, São Paulo State University (UNESP), Bauru/SP, SP 17033-360 Brazil; 4 Institute of Sciences and Engineering, São Paulo State University (UNESP), Itapeva/SP, 18409-010 Brazil

**Keywords:** Electronic structure calculations, Melanin-derivatives, Antibacterial effects, Density functional theory

## Abstract

**Context:**

Melanin-like dopamine oligomers are promising biomaterials due to their biocompatibility and metal-chelating ability. In this work, Zn^2+^-chelated melanin-related structures were investigated to clarify the effects of metal coordination on their electronic structure. The results indicate that Zn^2+^ coordination induces significant electronic polarization, modifies local reactivity patterns, and activates the coordination sites. The theoretical results are also consistent with available spectroscopic observations, providing molecular-level insight into the antibacterial behavior of these complexes and supporting the design of related bioactive materials.

**Methods:**

Density functional theory (DFT) calculations were performed for 5,6-dihydroxyindole (DHI), 5,6-dihydroxyindole-2-carboxylic acid (DHICA), and DHI-based oligomeric structures in different deprotonation states. Geometry optimizations and electronic-structure analyses were carried out using the B3LYP functional and 6-31G(d,p) basis set, including Grimme correction (GD3) and solvent effects through the polarizable continuum model (PCM) for water, as implemented in the Gaussian 16 software package. Local reactivity was investigated through condensed-to-atom Fukui indices (CAFIs) and molecular electrostatic potential (MEP) analyses. Zn^2+^ coordination and complex stability were further evaluated using calculations performed with the ORCA computational package and complementary automated interaction site screening (aISS) analyses followed by geometry optimizations with the GFN2-xTB approach implemented in the xTB package.

**Supplementary Information:**

The online version contains supplementary material available at https://doi.org/10.1007/s00894-026-06901-6.

## Introduction

Melanins are widely used in biomaterials design and hold significant potential for biomedical applications, including hemorrhage control through induction of the hemotactic process [1], controlled drug release [2], inflammation reduction [3], bactericidal activity [4], and potential roles in cancer therapy among others. These functionalities are primarily attributed to their high biocompatibility and to the presence of functional groups, which enable interactions with molecules, enzymes, and ions via hydrogen bonds, electrostatic attraction, and π-π interactions [5]. Another notable feature is their facile production process, typically achieved by auto-oxidation under alkaline conditions, allowing their integration into nanoparticles, thin films, and bulk materials [6].

These pigments are found in animals, plants, and cells, and their biological synthesis occurs through the enzymatic oxidation of aromatic compounds, resulting in three major classes of biological melanins: eumelanins, pheomelanins, and neuromelanins. Each class exhibits distinct physicochemical properties defined by its molecular constituents [7, 8]. In addition to natural biosynthesis, derivatives can also be obtained through synthetic routes, most commonly via the auto-oxidation of catecholamines. In basic aqueous solutions, catecholamines undergo a cascade of reactions that yield monomeric and oligomeric intermediates, which subsequently assemble into aggregates stabilized by both covalent and non-covalent interactions. This set of compounds is commonly referred to as polydopamine [9], although a more appropriate designation is dopamine-melanin, since there is insufficient evidence of effective covalent bond formation to characterize it as a polymer [10].

A relevant aspect in the use of dopamine-melanin in its various forms lies in the ability of these compounds to incorporate metal ions on their surface through covalent bonds, coordination bonds, and electrostatic attraction [9]. This enables the formation of diverse hybrid structures. Reports in the literature describe the incorporation of cerium, iron, copper, manganese, silver, and other metallic ions into melanin-like particles [8, 11–16], which are subsequently employed as building blocks in micro and nanoscale biocomposites. In particular, a recent study by Eliato et al. demonstrated the pronounced bactericidal activity of Zn^2+^-complexed dopamine-melanin particles. In their experiments, the system efficiently removed both gram-negative bacteria and endotoxins from human blood. Once the external membrane of these types of bacteria are negatively charged, as well as the released endotoxins, strong electrostatic interactions resulted in approximately 90% elimination of Escherichia coli and complete endotoxin removal in both phosphate-buffered saline (PBS) and human blood [17]. However, important gaps remain in elucidating the complexation mechanisms and the structural organization of these metal-chelated assemblies.

Here we present a comprehensive molecular-level computational investigation of representative dopamine-derived melanin oligomers, examined both in isolation and upon coordination with Zn^2+^. Using density functional theory (DFT)-based calculations and local reactivity descriptors, we elucidate how metal coordination reshapes the electronic structure, induces pronounced charge polarization, and activates specific reactive sites within melanin oligomers. Our results demonstrate that metal coordination strongly localizes reactivity on deprotonated oxygen atoms and on the metal center, promoting structural distortions and electronic rearrangements that substantially modify the electrostatic properties of the complexes. Furthermore, the predicted bond length variations and orbital redistributions are consistent with available experimental spectroscopic observations, providing a molecular-level interpretation of the electronic effects induced by metal ion coordination. Overall, this work establishes a theoretical framework for understanding Zn^2+^ coordination in dopamine-derived melanin oligomers and provides insights that may contribute to the interpretation and rational design of bioactive melanin-based materials, including systems involving alternative metal ions.

## Materials and methods

**Fig. 1:**
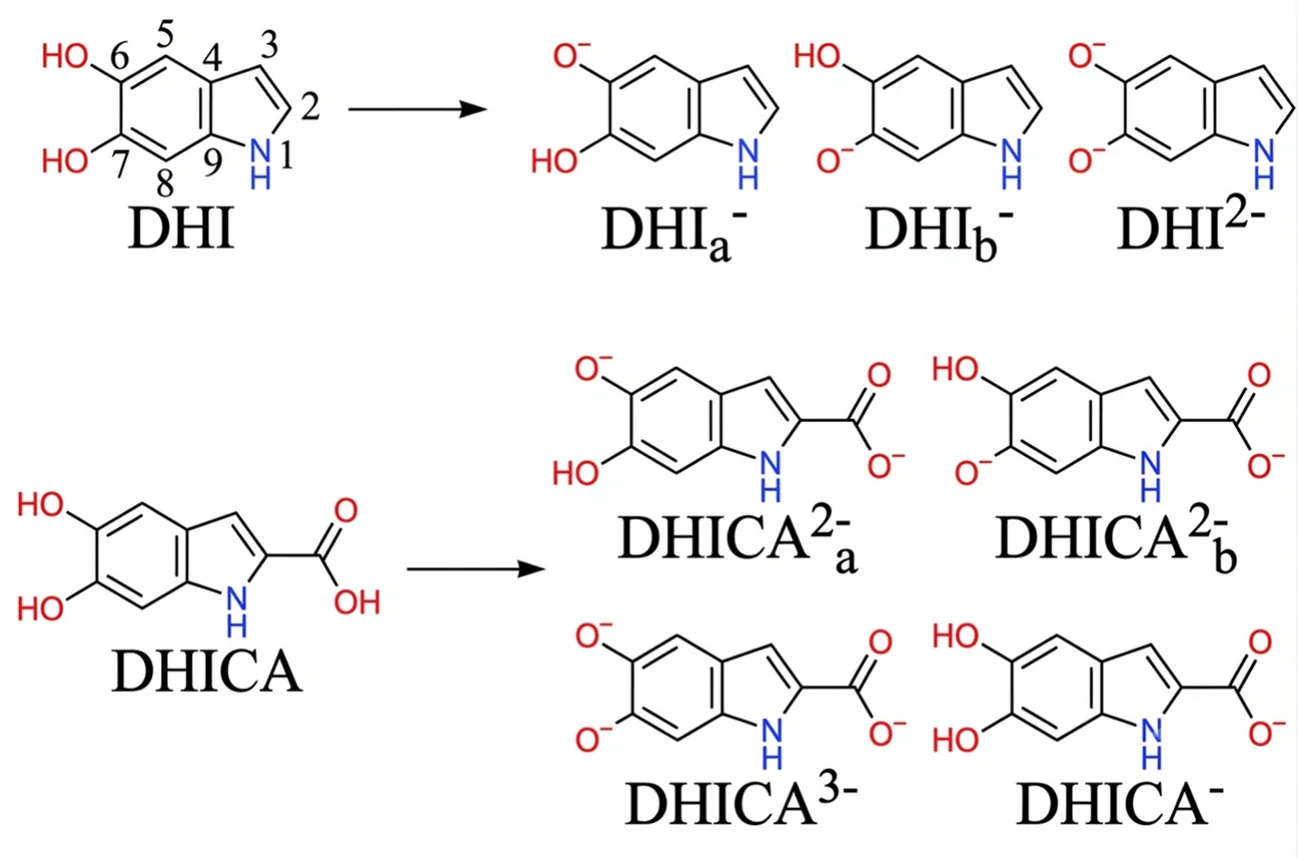
Melanin basic structures: DHI, DHICA and their deprotonated structures

Figure 1 illustrates the structures of the fundamental building blocks of melanin-based systems: 5,6-dihydroxyindole (DHI) and 5,6-dihydroxyindole-2-carboxylic acid (DHICA). DHICA-based structures are found in greater abundance in natural melanins, extracted from plants and animals, compared to those obtained through synthetic routes [18]. Both building blocks can exist in different oxidation states [19]. In particular, compounds with varying degrees of deprotonation were considered in order to simulate systems with distinct anchoring sites. For the DHICA monomer, the sequential deprotonation pathway was established based on the thermodynamic behavior of its functional groups, where the carboxylic acid group deprotonates at a significantly lower pH than the phenolic hydroxyl groups [20]. Consequently, to realistically simulate the activation of the catechol ring, any model featuring a deprotonated phenolic oxygen was systematically treated as a di-(DHICA_a_^−2^, DHICA_b_^−2^) or tri-deprotonated (DHICA^−3^) species, ensuring that the carboxylate group (–COO^−^) is already cleaved. Oligomeric systems composed of two DHI subunits were also investigated. Specifically, the 2–5′, 2–8′, 5–5′, and 5–8′ dimeric systems were evaluated once they have been proposed as the lowest energy DHI/DHICA oligomers [21]. These oligomeric models are expected to retain the essential coordination motifs and electronic features of melanin, providing a suitable framework for investigating the molecular-level effects of Zn^2+^ coordination. Our approach, however, is limited to local molecular interactions and does not explicitly account for the supramolecular organization of bulk melanin.

The geometries were fully optimized within a density functional theory (DFT) framework using the B3LYP hybrid exchange–correlation (XC) functional [22–25] coupled with Grimme’s D3 dispersion correction (GD3) [26] and the 6-31G(d,p) basis set for all the atoms [27–29]. This approach is consistent with previous studies from our group on related systems [19, 21, 30]. Solvent effects (water) were incorporated through the polarizable continuum model (PCM) [31]. All calculations were performed using the Gaussian 16 software package [32]. The net charge of each model was defined according to its degree of deprotonation and Zn^2+^ coordination. Accordingly, neutral, mono-, di-, and tri-deprotonated melanin models carried net charges of 0, −1, − 2, and − 3, respectively, whereas the corresponding Zn^2+^ complexes exhibited net charges of +1, 0, and − 1. Since Zn^2+^ is a closed-shell ion and the investigated models differ only by deprotonation, all systems were treated as singlet ground states (spin multiplicity = 1) (see Supplementary information for details). All the fully optimized Cartesian coordinates of calculated structures are provided in the Supplementary information.

The local reactivities of the compounds were estimated via condensed-to-atom Fukui indices (CAFIs) [33, 34] and molecular electrostatic potential (MEP) maps [35, 36]. Derived from conceptual DFT, CAFIs were used to identify the most reactive centers on the molecules for interactions with electrophiles (*f*^*−*^) and nucleophiles (*f*^+^). The Hirshfeld partitioning charge method [37] was employed to avoid negative indexes [38, 39]. MEP analyses were employed to identify electron-rich and electron-deficient molecular regions, which are crucial for predicting possible electrostatic interactions.

To investigate the coordination of Zn^2+^ by representative melanin-like dopamine oligomers, the melanin structures were optimized taking into account deprotonation processes. Subsequently, these structures were re-optimized in the presence of the metal ion attached to more reactive sites (identified via CAFI). Complexation energies were estimated to evaluate the chemical stability of metal-chelating systems, using the ORCA computational package [40], employing the same level of theory as used for geometry optimizations (B3LYP/6-31G(d,p)/GD3/PCM). ORCA was selected for this step due to its robust, native implementation of the counterpoise (CP) correction for basis set superposition error (BSSE) in the presence of implicit solvent models (PCM), which ensures highly accurate and solvent-corrected binding energies. Absolute consistency between the packages was guaranteed by employing identical functional, basis set, and solvation parameters. Additional complexation analyses were conducted via docking automated interaction site screening (aISS) method, with the resulting geometries optimized via tight binding DFT approach (GFN2-xTB) [41] as implemented in xTB computational package [42].

## Results and discussion

### Monomeric structures

**Fig. 2:**
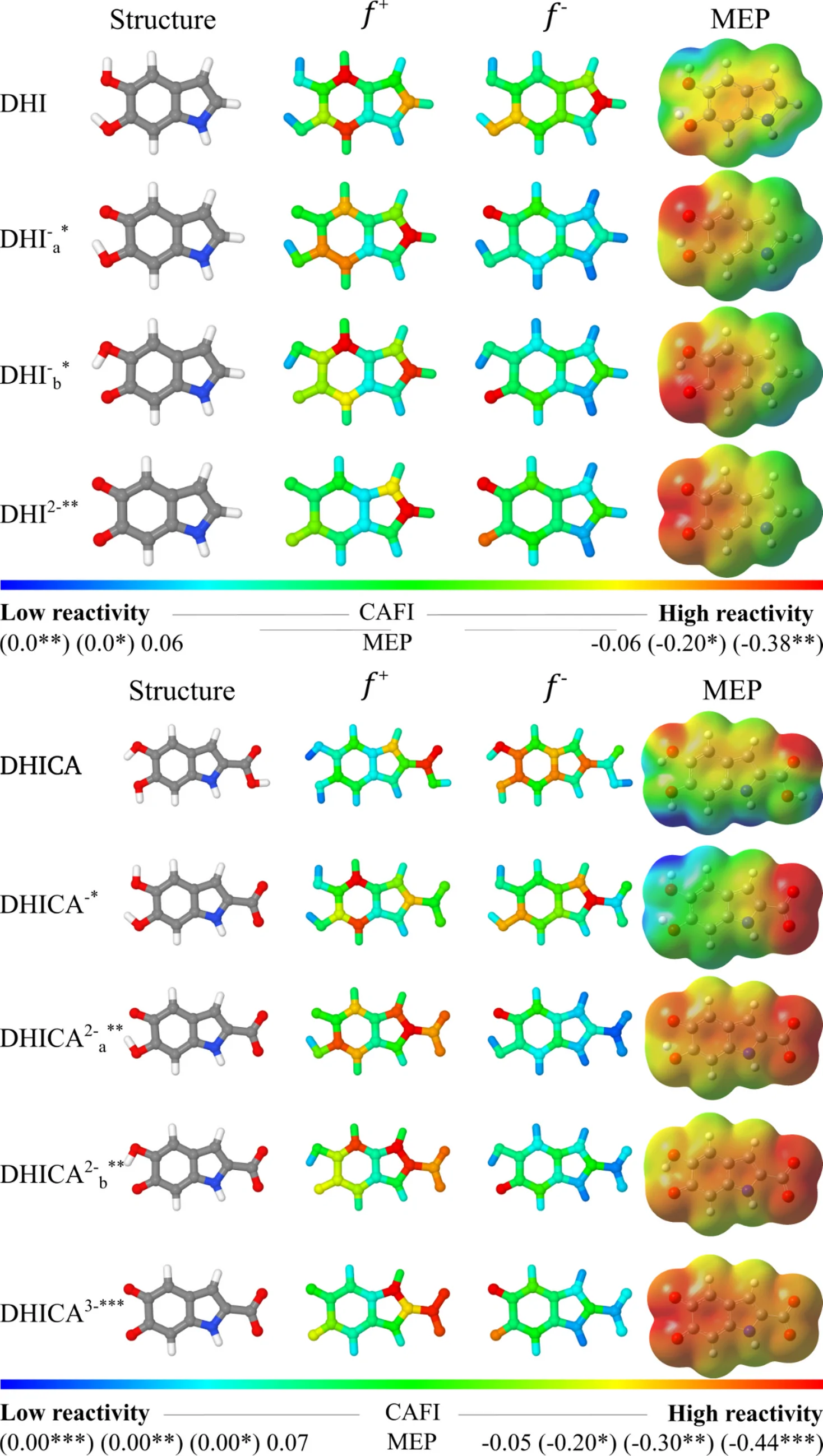
MEP and CAFI for DHI (**a**) and DHICA (**b**) units with distinct deprotonation degrees. The numerical scale of the electrostatic potential is expressed in atomic units (a.u.), while the condensed-to-atom Fukui indices (CAFIs) are dimensionless

Figure 2 displays color maps associated with CAFIs and MEPs for DHI and DHICA compounds. Red and blue regions indicate the position of reactive (negatively charged) and non-reactive (positively charged) molecular sites, respectively, for CAFI (MEP) representations. Intermediate colors denote sites with intermediate reactivity (net charge), following a blue-green–red (BGR) color scale. MEPs are presented in atomic units.

The MEPs of the distinct units reveal that the most negative regions are primarily localized on deprotonated oxygens and on the carbonyl oxygens of DHICA, whereas the most positive potentials are concentrated on hydrogen atoms, particularly those of –OH groups. Unmodified DHI presents high reactivity (CAFI values) on common polymerization sites, with *f*^−^ centered on *C*_2_ and *f*^+^ centered on *C*_5_ and *C*_8_ as reported elsewhere [21, 30]. Deprotonation induces high reactivity towards electrophiles on unprotected oxygens, while an intensification of local reactivity on *C*_2_ is noticed for *f*^+^. The local reactivity on DHICA suggests the plausibility of COOH release, as stated in other work [21]. Deprotonation of quinone drives the highest *f*^−^ relative reactivity towards the unprotected oxygens. Distinct effects are noticed for COOH deprotonation, with activation of *C*_5_, *C*_8_, and *C*_3_ polymerization sites for *f*^+^ and *C*_2_ for *f*^−^.

**Fig. 3:**
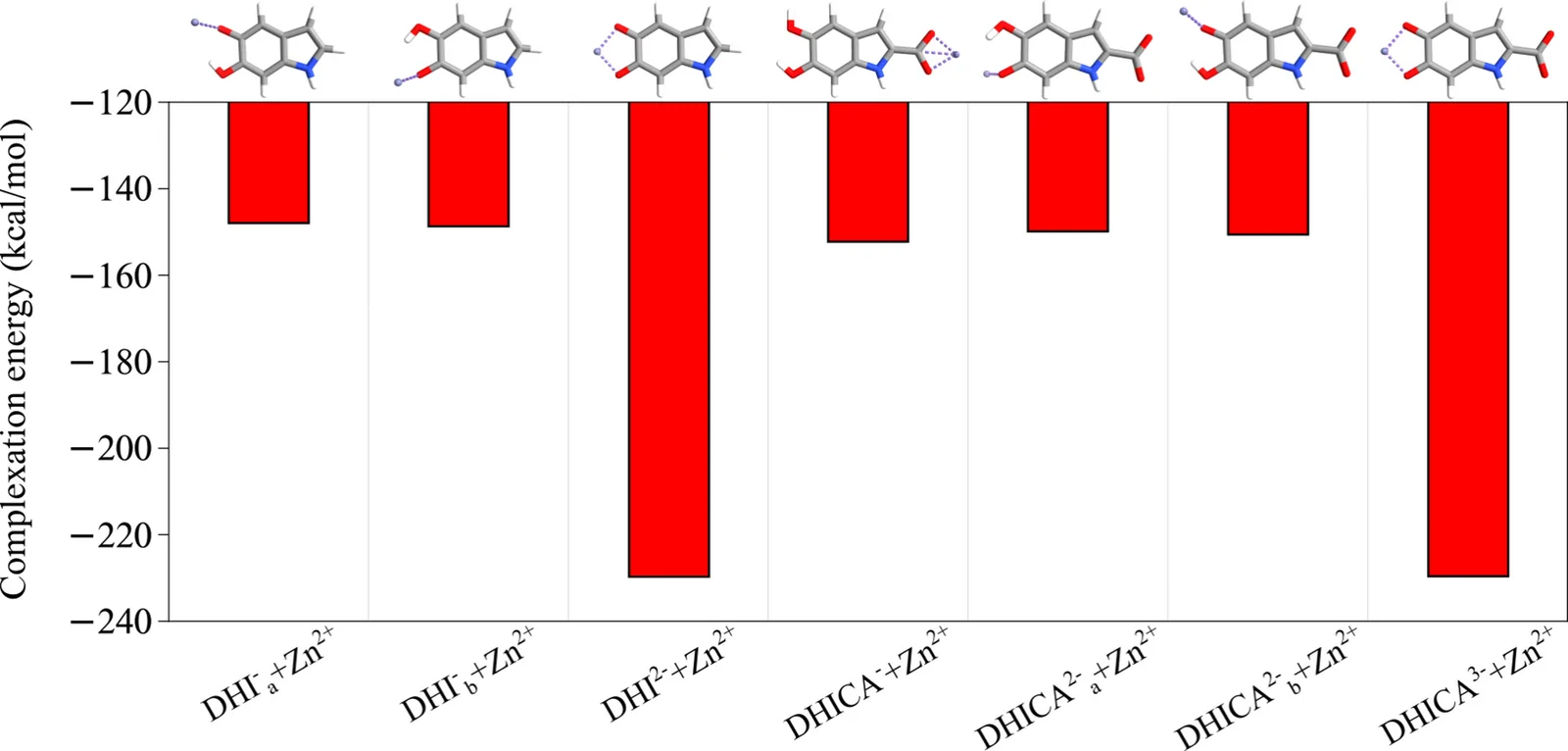
Optimized structures and binding energies of the deprotonated melanin building blocks after Zn^2+^ complexation

The reactivity data suggest a higher likelihood of chelation between the compounds and the metal ions through the deprotonated oxygen atoms in both DHI and DHICA [8]. To further clarify this behavior, metal-melanin complexation energies were estimated considering different melanin protonation states with a divalent zinc ion (Zn^2+^), given the well-known ability of the metal to form stable complexes with chelating groups and organic frameworks [43]. For these calculations, the ion was initially positioned at a distance of 2.2 Å from both oxygen atoms, followed by full geometry optimization. In the case of a single deprotonated hydroxyl group, only one configuration was considered, as the others were very similar. Figure 3 shows the optimized structure and the corresponding complexation energies for selected systems.

The high complexation energies suggest that the melanin-dopamine systems possess a strong affinity to interact with the zinc ion. Since the dideprotonated systems exhibited significantly higher absolute values, they were selected for the subsequent studies. These results are also compatible with the higher affinity of Zn^2+^ to hydroxyl oxygen atoms in relation to COO^−^ ones [20].

### Dimeric structures

**Fig. 4:**
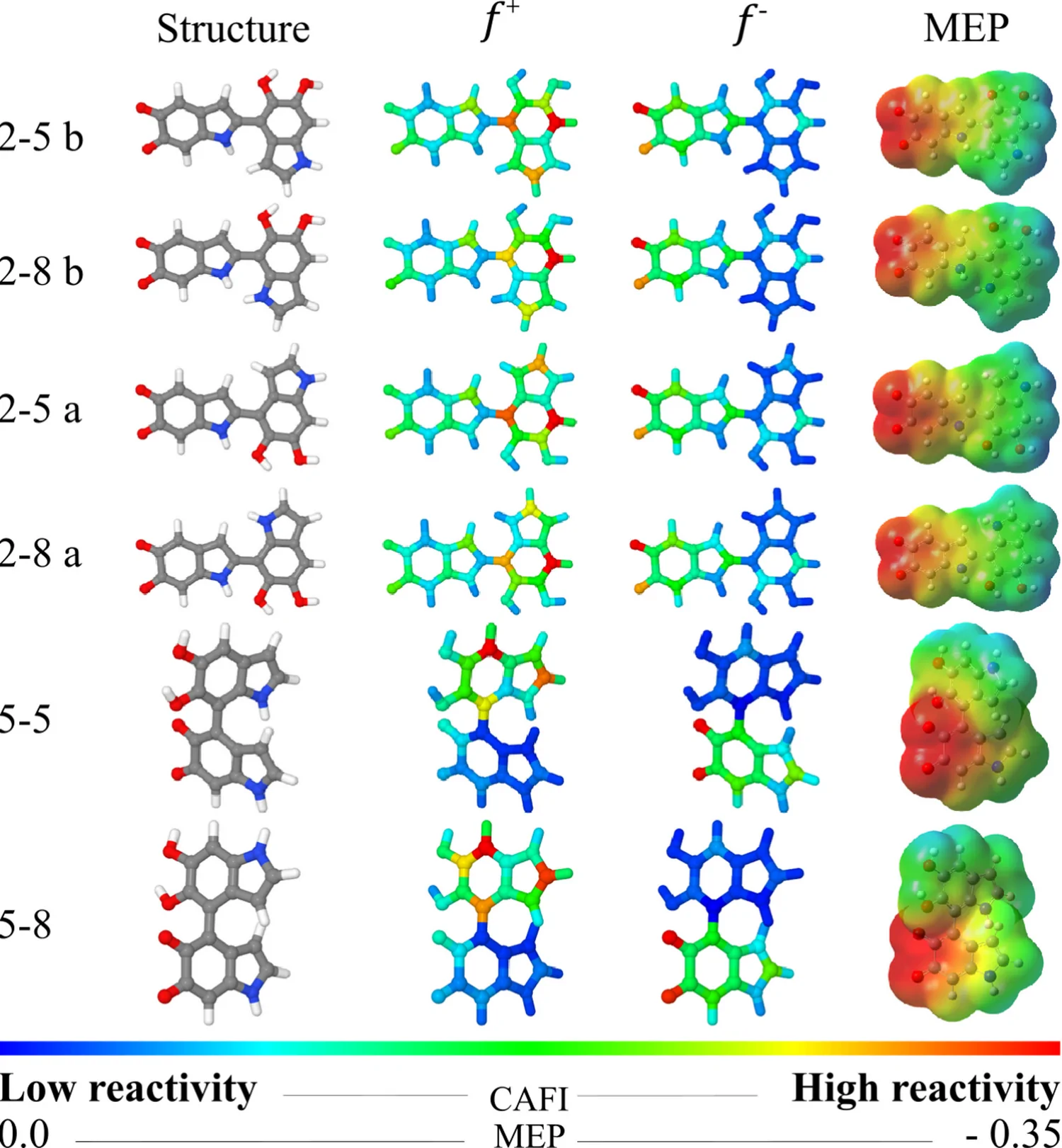
MEP and CAFI for different DHI-based melanin dimeric structures. The numerical scale of the electrostatic potential is expressed in atomic units (a.u.), while the condensed-to-atom Fukui indices (CAFIs) are dimensionless

Considering the oligomeric nature of melanins, 2–5′, 2–8′, 5–5′, and 5–8′ dimeric systems were considered to identify the influence of oligomerization on the local reactivity and electronic properties of the systems. Only DHI-based oligomers were evaluated given the enhanced melanin-Zn^2+^ interaction reported for synthetic (DHI-rich) melanin systems [17, 44]. Figure 4 shows the CAFIs and MEPs obtained (the same definitions used in Fig. 2 are applied).

The reactivity pattern noticed for monomers remains in oligomeric systems. Deprotonated units dominate the reactivity toward electrophiles (*f*^−^) while protonated ones play a major role in the reactivity toward nucleophiles (*f*^+^). These features concentrate again the highest *f*^−^ values on deprotonated oxygens and drive the highest *f*^+^ to *C*_5_/*C*_8_ common polymerization sites. Complementarily, MEPs indicate high electron density on deprotonated oxygens.

An integrated analysis of CAFI and MEP results provides robust evidence for the interaction between the Zn^2+^ ion and the deprotonated oxygen sites. This coordination is shown to be plausible through a dual-regime perspective: MEP maps validate the favorability of hard-hard electrostatic interactions, while CAFI analysis underscores the contribution of soft–soft interactions mediated by frontier orbital coupling.

**Fig. 5:**
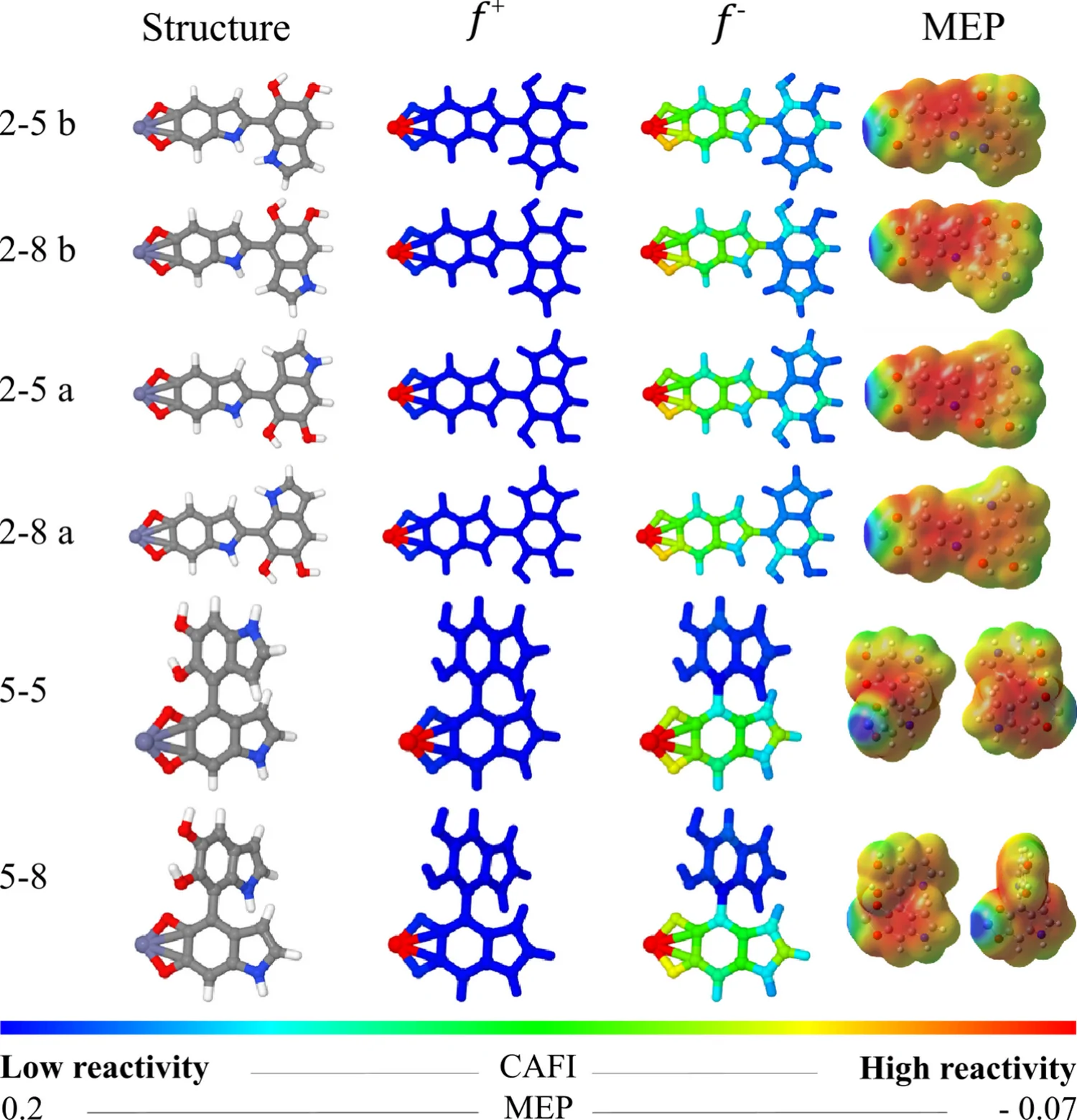
MEP and CAFI for different dimers of DHI + Zn^2+^.The numerical scale of the electrostatic potential is expressed in atomic units (a.u.), while the condensed-to-atom Fukui indices (CAFIs) are dimensionless

This trend is reinforced by molecular docking simulations conducted via aISS/GFN2-xTB approach to identify stable structures from multiple initial positions. For each system, the top 15 poses, corresponding to the lowest energy, were selected for further geometry optimization. The resulting low-energy complexes were found to be structurally compatible with the initial hypothesis, strongly supporting the proposed binding mode (the complete set of structures are presented in the Supplementary Information). Based on these results, Zn^2^⁺ ions were positioned near the oxygen atoms of the deprotonated dimer unit; the resulting structures were then fully optimized. Figure 5 presents the CAFI and MEP analyses of the optimized structures.

Regarding interaction with nucleophiles (*f*^+^), the local reactivity is centered on the metal ion, while the rest of the structure remains largely inert. High reactivity is also observed for interactions towards electrophiles (*f*^−^), with intermediate reactivity along the main chain, following the same pattern identified for the isolated DHI^−2^ unit. The MEP reveals a region with relatively more positive charge on the metal, a finding that aligns with reported experimental studies [17] and is correlated with the most reactive site toward nucleophiles (*f*^+^). This localized electropositive character is crucial for the compound’s activity against Gram-negative bacteria. The outer membrane of these microorganisms is characterized by a high density of lipopolysaccharides (LPS), which imparts a strong negative net charge to the cell surface. Consequently, the metal-centered positive potential promotes a directed electrostatic attraction toward the bacterial membrane (and potentially toward released LPS aggregates), facilitating membrane docking and subsequent penetration or disruption mechanisms [17].

**Fig. 6:**
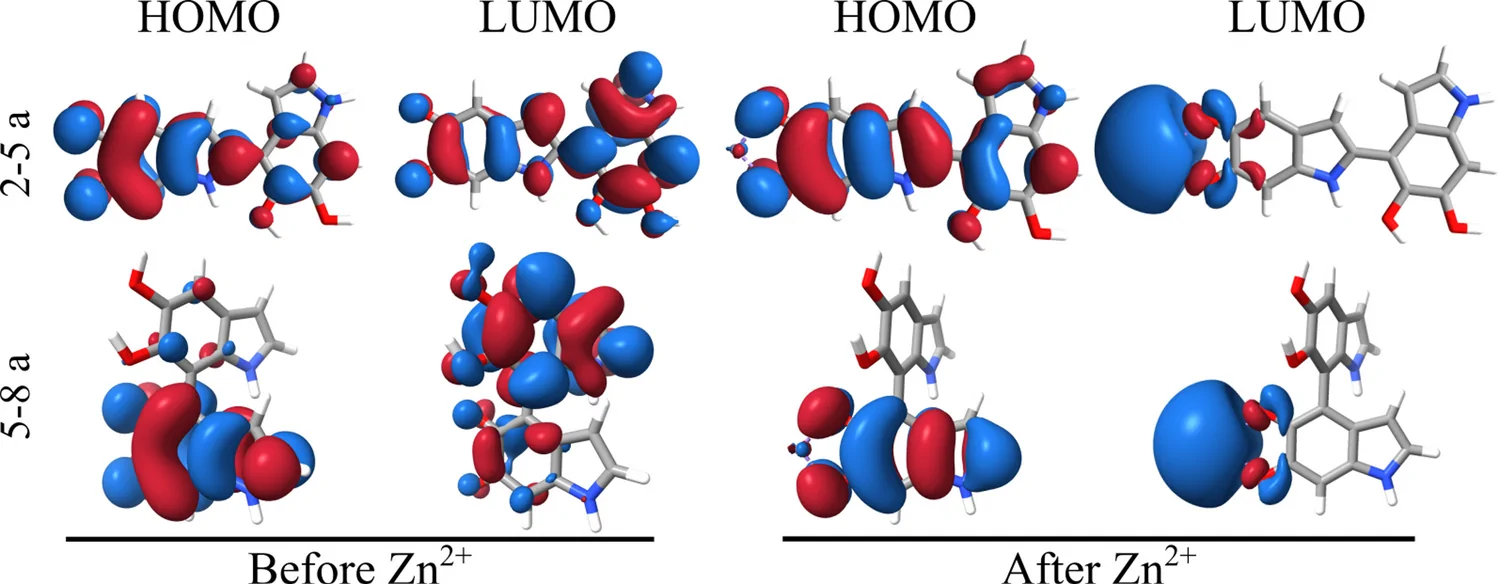
Frontier molecular orbitals for selected dimers with and without Zn^2+^

To identify the effect of the metal on the electronic structure of the system, an analysis of the spatial distribution of frontier Kohn–Sham-based molecular orbitals (KS-FMOs) was performed. Figure 6 presents the highest occupied and lowest unoccupied KS-FMOs (HOMO and LUMO) for the 2–5′ and 5–8′ dimers, before and after metal chelation (similar results are obtained for 2–8′ and 5–5′).

Note that HOMO spreads out across both units in non-chelated 2–5′ and 5–8′ molecules, with a more pronounced contribution on the deprotonated moiety. An opposite effect is noticed for the LUMO, which is dominated by the protonated unit. A significant change is noticed after ion incorporation: (i) the HOMO shifts to the chelated unit, especially for the 5–8′ dimer; and (ii) the ion dominates the LUMO distribution with significant changes in the orbital symmetry.

These results align with the CAFI, once *f*^+^ index reflects molecular sites that are prone to accept electrons, which is correlated with LUMO distribution (dominated by Zn^2+^ ion). Conversely, a more uniform *f*^*−*^is consistent with the HOMO distribution.

**Fig. 7:**
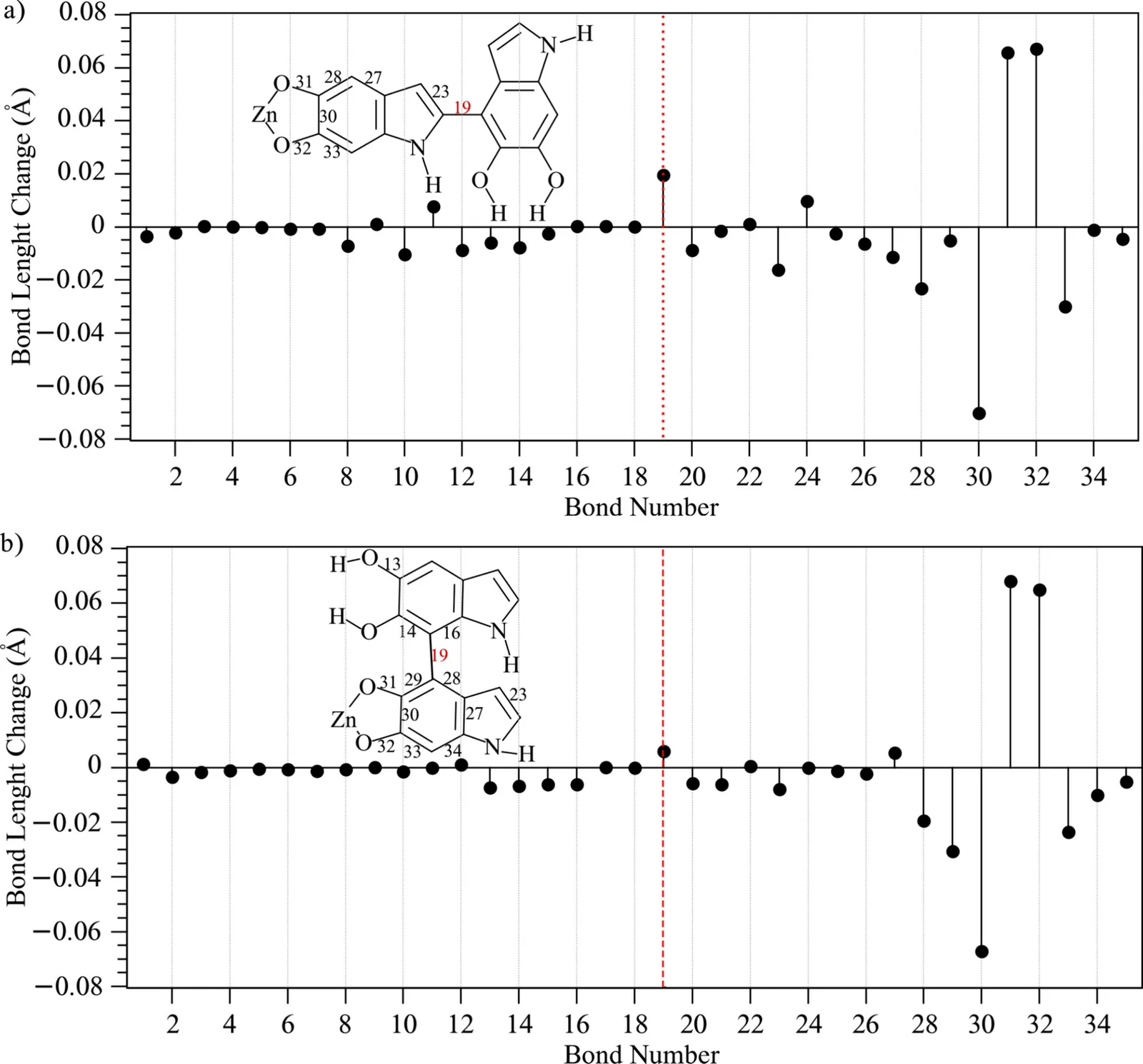
Bond length changes induced on **a)** 2–5′ and **b)** 5–8′ dimers by Zn^+2^ ion complexation

Eliato et al. [17], using peak shifts in the deconvolved Raman spectra, showed that the formation of Zn-melanin bonds alters the carbon–carbon bond lengths between adjacent melanin units. To compare with these experimental findings, we calculated the bond length changes in melanin models with and without the Zn^2^⁺ ion. Figure 7 presents the calculated bond length alterations upon ion complexation for the 2–5'a and 5–8'a structures.

In both dimeric structures it is noticed an influence of the ion on the oxygen bonds. Specifically, an increase in bonds 31 and 32 is observed followed by reduction in bond 30, suggesting a decrease in the electron density in C–O bonds and an increase in *C*_6_–*C*_7_ one. This effect decays with the distance from the ion, causing minor perturbations to the distal sites. Nevertheless some inductive effect is noticed on bond 19 (that connects adjacent monomers in 2–5′ dimers) with a significant increase (considering its relative distance to Zn^2+^), consistent with results reported in ref. [17]. Although the 5–8′ dimer also shows an increase in bond 19 length, the effect is very small (similar results were found for the 5–5′). Given the dominance of 2–5′ connections in DHI-rich systems and 5–5′/5–8′ connections on DHICA-rich ones, this result suggests a distinct response of DHI and DHICA structures to the ion coordination, which may serve as spectroscopic markers for characterizing these oligomeric systems, supporting the dominance of the 2–5′ and 2–8′-based structures in ref. [17].

## Conclusion

DFT-based calculations were conducted to provide a comprehensive molecular-level understanding of the interaction between Zn^2+^ ions and melanin-like dopamine oligomers.

Our findings reveal that metal chelation is a highly favorable process, particularly at deprotonated oxygen sites, resulting in significant structural and electronic rearrangements. The local reactivity analysis demonstrates that the incorporation of the metal center induces a pronounced electronic polarization. While the organic framework remains relatively inert, the Zn^2+^ center emerges as the primary site for nucleophilic attacks (*f*^+^ ) and concentrates a high positive electrostatic potential. This specific localized electropositivity provides a theoretical foundation for the observed bioactivity of Zn^2+^-chelated melanin-like systems against Gram-negative bacteria [17]. Furthermore, our electronic structure analysis showed that the LUMO distribution is dominated by the metal ion after complexation, confirming the shift in the system’s chemical responsiveness. The bond length variations, particularly observed in the 2–5′ DHI-rich dimers, agree with experimental spectroscopic markers reported in the literature, validating our models.

These findings help elucidate how ion-induced charge polarization influences the local reactivity of melanin-like compounds, providing a basis for assessing the role of electronic structure in the bioactivity of these metal-chelated systems.

## Supplementary Information

Below is the link to the electronic supplementary material.

ESM 1 PDF (1.21 MB)

## Data Availability

No datasets were generated or analyzed during the current study.
