# Supplementary material for: Zn2+-induced electronic polarization and local reactivity in melanin-like dopamine oligomers: a DFT study
Source: J Mol Model. 2026 Aug 20;32(9):324. doi: 10.1007/s00894-026-06901-6 (PMC13493412; doi:10.1007/s00894-026-06901-6)
Supplement: Supplementary file 1 [file 894_2026_6901_MOESM1_ESM.pdf]

SUPPLEMENTARY MATERIAL

Zn^{2+}-induced electronic polarization and local reactivity in melanin-like dopamine oligomers: A DFT study

João Pedro Dionizio [a], Henrique Rodrigues Souza-Silva [a], Jean Valdir Uchôa Teixeira [a], Orisson Ponce Gomes [b], Didier Bégué [b], Paulo Noronha Lisboa-Filho [c], Augusto Batagin-Neto [d,*]

*email: a.batagin@unesp.br

[a] São Paulo State University (UNESP), School of Sciences, POSMAT, Bauru/SP 17033-360, Brazil

[b] Université de Pau et des Pays de l'Adour, E2S UPPA, CNRS, Institut des Sciences Analytiques et Physico-chimie pour l'environnement et les matériaux (IPREM), 64000 Pau, France

[c] São Paulo State University (UNESP), School of Sciences, Department of Physics and Meteorology, Bauru/SP 17033-360, SP, Brazil

[d] São Paulo State University (UNESP), Institute of Sciences and Engineering, Itapeva/SP 18409-010, Brazil

Summary

S1. Charge and spin configuration of the systems.. S2

S2. Complexation studies via docking.. S2

S3. Cartesian coordinates of optimized geometries... S4

S1. Charge and spin configuration of the systems

The primary building blocks of the melanin-like models, namely 5,6-dihydroxyindole (DHI) and 5,6-dihydroxyindole-2-carboxylic acid (DHICA), were evaluated in their neutral, mono-, di-, and tri-deprotonated states. The net charges of these metal-free species were assigned as 0, -1, -2, and -3, respectively. Upon coordination with the divalent zinc cation Zn^{2+} (which carries a formal +2 charge), the net charges of the resulting complexes were adjusted accordingly to +1, 0, and -1. Because all organic ligands are closed-shell molecules and the Zn^{2+} ion possesses a completely filled d^{10} electronic configuration, all isolated precursors and their corresponding metal complexes were treated in their closed-shell singlet ground states (spin multiplicity M = 1). The dimeric structures investigated in this work follow the same protonation and coordination configurations established for their respective monomeric units. The complete list of net charges and spin multiplicities for each calculated system is summarized in Table S1.

Table S1. Computational parameters (net charge and spin multiplicity) specified for the isolated melanin monomers and their corresponding Zn^{2+} complexes. The dimers follow the configuration of their corresponding deprotonated/complexed monomer units.

Net charge	Spin multiplicity	Systems
-3	1	$DHICA^{-3}$
-2	1	DHI^{-2}_{x} and $DHICA^{-2}_{x}$
-1	1	DHI^{-1}_{x}, $DHICA^{-1}_{x}$, and $DHICA^{-3}+Zn^{2+}$
0	1	DHI, DHICA, $DHI^{-2}_{x}+Zn^{2+}$, and $DHICA^{-2}_{x}+Zn^{2+}$
+1	1	$DHI^{-1}_{x}+Zn^{2+}$ and $DHICA^{-1}_{x}+Zn^{2+}$

Note: x = a or b, depending on the deprotonated hydroxyl site.

S2. Complexation studies via docking

Figure S1 presents the 15 final structures obtained from the docking procedure for each dimer in the presence of the zinc ion in aqueous environment, as well as their corresponding energy values. The calculations were performed employing the GFN2-xTB method, as implemented in xTB computational package. Initial configurations were generated by exploring different dimer/ion relative positions, followed by geometry optimization. The resulting structures were ranked according to their total energies, and the lowest-energy configurations were selected for analysis.

The points in the graph represent the energy of each structure, from lowest to highest energy. The first structure corresponds to the lowest energy compound for each dimer. The lowest-energy structures correspond to configurations where the zinc ion is coordinated by both oxygen atoms; consequently, these conformers were prioritized for subsequent optimization and analysis.

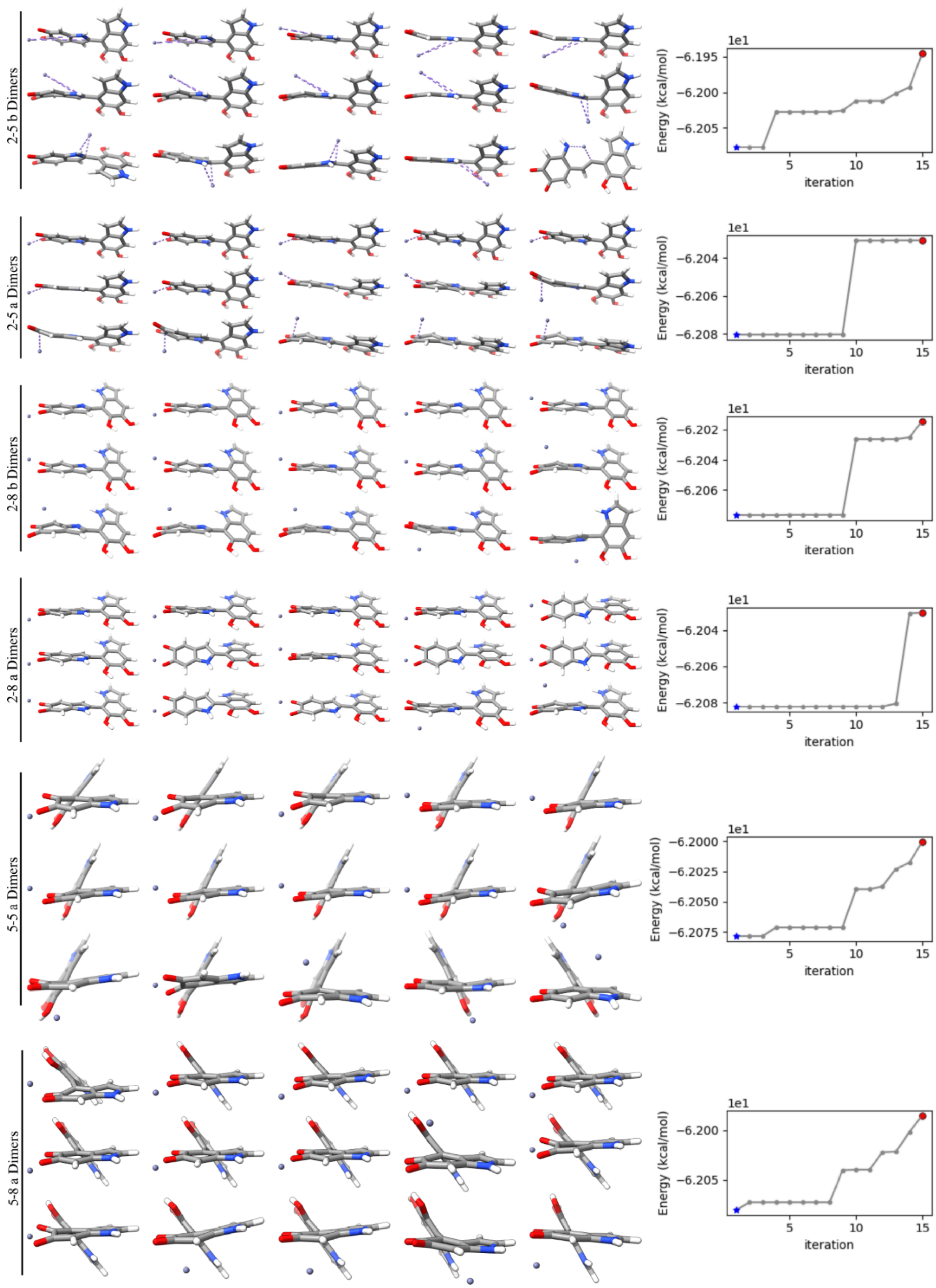

Figure S1. Docking studies conducted via tight binding DFT approach (GFN2-xTB).

S3. Cartesian coordinates of optimized geometries

To ensure the computational reproducibility of this study, the fully optimized Cartesian coordinates of all calculated monomers, dimers, and Zn^{2+} complexes are provided below.

Monomeric Structures

DHI$^{-}_{a}$

C	0.91943	0.66146	-0.00011
C	0.92966	-0.75745	0.00007
C	-0.31541	-1.44743	0.00021
C	-1.47028	-0.70123	0.00015
C	-1.50137	0.76196	-0.00004
C	-0.26229	1.43130	-0.00017
C	3.07915	-0.02997	-0.00001
C	2.30754	-1.16828	0.00001
H	-0.36065	-2.53421	0.00033
H	-0.23293	2.51839	-0.00033
H	2.68075	-2.18439	0.00009
N	2.24127	1.07587	-0.00018
H	2.54775	2.03525	-0.00050
O	-2.68600	1.29542	-0.00011
O	-2.73179	-1.24209	0.00021
H	-3.25250	-0.38832	0.00009
H	4.15245	0.09337	0.00001

DHI$^{-}_{a}$+Zn^{2+}

C	1.85108	-0.70564	-0.17273
C	2.23539	0.62571	0.15252
C	1.25922	1.64339	0.13392
C	-0.04242	1.30932	-0.19677
C	-0.41781	-0.03263	-0.51204
C	0.53345	-1.04740	-0.50377
C	4.05734	-0.70698	0.28167
C	3.64313	0.59350	0.43741
H	1.50547	2.67382	0.37096
H	0.23912	-2.06417	-0.74342
H	4.26710	1.42914	0.72179
N	2.98288	-1.49248	-0.08693
H	3.02065	-2.48407	-0.26190
O	-1.71310	-0.25105	-0.87249
O	-1.02236	2.27231	-0.20792
H	-1.80238	1.84429	-0.59993
H	5.03706	-1.14548	0.40426
Zn	-2.99932	-0.53506	0.38796

**DHI^-_b**			
C	0.92394	-0.66413	0.00006
C	0.92529	0.75316	-0.00008
C	-0.30698	1.46195	-0.00010
C	-1.52180	0.75956	-0.00001
C	-1.44892	-0.70124	0.00018
C	-0.27146	-1.41865	0.00021
C	3.07743	0.01467	-0.00025
C	2.30756	1.15685	-0.00011
H	-0.32201	2.55016	-0.00017
H	-0.27115	-2.50553	0.00030
H	2.68535	2.17121	-0.00017
N	2.24484	-1.08718	-0.00001
H	2.55026	-2.04680	0.00029
O	-2.69136	-1.27229	0.00007
O	-2.73251	1.24679	0.00006
H	-3.22695	-0.42030	-0.00021
H	4.15111	-0.10747	-0.00039

**$DHI^-_b + Zn^{2+}$**			
C	-2.20645	-0.54977	-0.13128
C	-1.85090	0.79070	0.18912
C	-0.51080	1.08114	0.51432
C	0.42589	0.05649	0.51915
C	0.03102	-1.28048	0.20551
C	-1.27655	-1.59949	-0.12503
C	-4.06191	0.71960	-0.28166
C	-3.05281	1.57406	0.08617
H	-0.19068	2.09045	0.75581
H	-1.54956	-2.62202	-0.36374
H	-3.15205	2.63589	0.26272
N	-3.55589	-0.56069	-0.41445
H	-4.09125	-1.37316	-0.67649
O	0.99264	-2.25671	0.21968
O	1.73091	0.24754	0.87379
H	1.78075	-1.83306	0.60166
H	-5.10898	0.91321	-0.46387
Zn	3.01432	0.51445	-0.39402

DHI^{2-}

C 0.88545 -0.65519 -0.00008
C 0.88992 0.75818 0.00001
C -0.36766 1.43780 -0.00004
C -1.60869 0.75883 -0.00014
C -1.57899 -0.76304 0.00023
C -0.31197 -1.40487 0.00002
C 3.05646 0.02499 -0.00000
C 2.27115 1.16352 0.00003
H -0.39648 2.53109 -0.00010
H -0.29073 -2.49646 0.00008
H 2.64842 2.18046 0.00008
N 2.21181 -1.07845 -0.00024
H 2.51318 -2.03911 0.00001
O -2.68607 -1.42326 0.00061
O -2.75158 1.36711 -0.00042
H 4.13017 -0.09894 0.00001

DHI^{2-} +Zn^{2+}

C -2.00244 -0.66479 0.00668
C -2.01611 0.75326 0.00783
C -0.78333 1.44483 0.02431
C 0.41498 0.74019 0.03785
C 0.40334 -0.71772 0.03956
C -0.80909 -1.40520 0.02351
C -4.15728 0.00993 -0.03463
C -3.39797 1.15548 -0.01725
H -0.75010 2.53156 0.02211
H -0.80797 -2.49161 0.02005
H -3.77942 2.16780 -0.02410
N -3.31961 -1.09118 -0.01850
H -3.62236 -2.05171 -0.03041
O 1.57925 -1.38305 0.05060
O 1.60797 1.38380 0.04401
H -5.22989 -0.11857 -0.05797
Zn 2.86722 -0.01004 -0.03614

DHICA^{-}

C 0.09277 0.62198 -0.00030
C 0.13495 -0.80478 0.00000
C 1.39199 -1.44625 -0.00012
C 2.53901 -0.67105 -0.00021
C 2.47512 0.74954 -0.00007
C 1.25437 1.40770 -0.00027
C -2.03410 -0.14082 -0.00006
C -1.22493 -1.25824 -0.00001
H 1.46751 -2.53082 -0.00024
H 1.22418 2.49208 -0.00005
H -1.57480 -2.28040 0.00005
N -1.23143 0.98577 -0.00028
H -1.63353 1.91135 -0.00013
O 3.63806 1.47768 0.00040
O 3.82576 -1.17338 -0.00101
C -3.53692 0.02258 0.00010
O -4.21900 -1.03550 0.00015
O -3.94418 1.22080 0.00025
H 3.80421 -2.13926 0.00676
H 4.37383 0.84585 0.00292

DHICA^{-}+Zn^{2+}

C -1.44142 -0.64353 -0.04578
C -1.48351 0.78346 -0.04154
C -2.73539 1.44087 -0.00685
C -3.87895 0.67329 0.02430
C -3.81824 -0.75704 0.01847
C -2.60695 -1.42555 -0.01801
C 0.67109 0.11565 -0.07891
C -0.13864 1.23867 -0.06395
H -2.79612 2.52521 -0.00030
H -2.58399 -2.50917 -0.01947
H 0.21455 2.25903 -0.06128
N -0.12449 -1.02058 -0.07133
H 0.23589 -1.96267 -0.06865
O -4.97940 -1.46798 0.05173
O -5.16080 1.16572 0.06426
C 2.11309 0.04088 -0.05914
O 2.80995 1.11979 -0.04607
O 2.71828 -1.09237 -0.03231
H -5.15237 2.13223 0.07984
H -5.71432 -0.83430 0.08033
Zn 4.44924 -0.03559 0.06054

$DHICA^{2-}_a$

C	0.13654	0.61523	0.00000
C	0.16863	-0.80942	-0.00013
C	1.43568	-1.46054	-0.00011
C	2.56778	-0.68075	0.00005
C	2.55539	0.78309	0.00018
C	1.29774	1.41644	0.00015
C	-2.00942	-0.14086	-0.00014
C	-1.19284	-1.25692	-0.00028
H	1.51446	-2.54565	-0.00021
H	1.23869	2.50249	0.00024
H	-1.54417	-2.28025	-0.00042
N	-1.19199	0.98260	-0.00006
H	-1.58513	1.91099	0.00007
O	3.72476	1.35073	0.00030
O	3.84580	-1.18375	0.00009
C	-3.50128	0.02496	-0.00009
O	-4.19863	-1.02885	-0.00011
O	-3.91807	1.22453	0.00007
H	4.33988	-0.31440	0.00020

$DHICA^{2-}_a + Zn^{2+}$

C	-0.96194	-0.35496	-0.25681
C	-1.15566	1.02960	0.03816
C	-0.03399	1.88559	0.05190
C	1.21456	1.35554	-0.22165
C	1.39800	-0.03348	-0.50339
C	0.30398	-0.89379	-0.52143
C	-3.17098	-0.01292	0.07614
C	-2.56149	1.21393	0.24552
H	-0.13005	2.94692	0.26061
H	0.45161	-1.94636	-0.74143
H	-3.07298	2.13496	0.48531
N	-2.20098	-0.95063	-0.22482
H	-2.44411	-1.91571	-0.39139
O	2.65126	-0.45739	-0.82617
O	2.32487	2.17041	-0.20769
C	-4.61710	-0.44499	0.15984
O	-5.45737	0.44949	0.44072
O	-4.82267	-1.67329	-0.06544
H	3.03287	1.66102	-0.63380
Zn	3.91695	-0.75358	0.44845

DHICA$^{2-}{}_{b}$

C	-0.13061	-0.61311	0.00002
C	-0.17439	0.80914	-0.00006
C	-1.43176	1.47568	0.00002
C	-2.62251	0.73477	0.00017
C	-2.50322	-0.72317	0.00026
C	-1.30349	-1.40307	0.00018
C	2.00796	0.13373	-0.00019
C	1.19118	1.25233	-0.00018
H	-1.48338	2.56305	-0.00003
H	-1.26963	-2.48957	0.00022
H	1.54508	2.27473	-0.00024
N	1.19800	-0.98687	-0.00014
H	1.59197	-1.91495	-0.00006
O	-3.72771	-1.33181	0.00038
O	-3.85058	1.18104	0.00029
C	3.50278	-0.03068	-0.00022
O	4.19634	1.02452	-0.00044
O	3.91974	-1.22927	-0.00014
H	-4.28800	-0.49491	0.00035

DHICA$^{2-}{}_{b}$ +Zn^{2+}

C	1.15911	-0.89688	0.00011
C	0.83675	0.45219	-0.34448
C	-0.51023	0.78553	-0.59732
C	-1.48369	-0.19772	-0.49597
C	-1.12890	-1.53580	-0.14803
C	0.18485	-1.90236	0.10082
C	3.07669	0.30147	-0.00846
C	2.07087	1.18348	-0.34302
H	-0.80693	1.79813	-0.85747
H	0.42983	-2.92538	0.36704
H	2.21007	2.23310	-0.55873
N	2.51765	-0.94882	0.19413
H	3.09016	-1.73915	0.45095
O	-2.14233	-2.45212	-0.05235
O	-2.81039	0.00606	-0.77272
C	4.56709	0.48237	0.16102
O	5.01926	1.64210	-0.02917
O	5.20145	-0.56556	0.47986
H	-2.93980	-1.95355	-0.31349
Zn	-3.81287	0.93837	0.41999

DHICA^{3-}

C	-0.17549	-0.60901	0.00002
C	-0.21585	0.81148	0.00000
C	-1.49581	1.44966	0.00009
C	-2.71375	0.73254	0.00019
C	-2.63635	-0.78994	0.00022
C	-1.34980	-1.39274	0.00012
C	1.98116	0.14029	-0.00018
C	1.14532	1.25560	-0.00009
H	-1.55652	2.54170	0.00008
H	-1.29408	-2.48298	0.00015
H	1.49530	2.28169	-0.00012
N	1.15638	-0.98204	-0.00013
H	1.54575	-1.91133	0.00005
O	-3.72306	-1.48115	0.00033
O	-3.87644	1.30099	0.00028
C	3.46023	-0.02324	-0.00025
O	4.17186	1.02878	-0.00037
O	3.89226	-1.22393	-0.00024

DHICA^{3-}+Zn^{2+}

C	-1.01055	-0.59819	0.03122
C	-0.99104	0.82544	0.03243
C	0.25977	1.48602	0.03720
C	1.44088	0.75403	0.04011
C	1.39576	-0.70346	0.04967
C	0.16793	-1.36309	0.04253
C	-3.15591	0.12739	0.00153
C	-2.35967	1.25626	0.01583
H	0.31944	2.57187	0.03182
H	0.14540	-2.44938	0.04164
H	-2.72637	2.27344	0.01187
N	-2.33266	-0.98631	0.01302
H	-2.71726	-1.91849	-0.00178
O	2.55745	-1.39582	0.05884
O	2.64960	1.37096	0.02769
C	-4.64771	-0.05575	-0.02887
O	-5.34438	0.97863	-0.03360
O	-5.04282	-1.24691	-0.04806
Zn	3.87173	-0.05333	-0.05145

Dimeric Structures

2-5(b)

N	0.000000	0.000000	0.000000
C	0.000000	0.000000	1.381205
C	1.297873	0.000000	1.828656
C	2.160963	0.001967	0.672292
C	3.588521	-0.020289	0.502818
C	4.037030	-0.110616	-0.834633
C	3.148047	-0.146788	-1.928513
C	1.766838	-0.089108	-1.780428
C	1.301624	-0.013676	-0.459410
O	5.392693	-0.129419	-1.106979
O	3.771171	-0.236674	-3.163984
N	4.108087	0.607001	2.854245
C	4.507230	0.075198	1.610742
C	5.842196	-0.325585	1.762591
C	6.280690	-0.011650	3.078104
C	7.498060	-0.183288	3.802517
C	7.665225	0.275861	5.127985
C	6.487141	0.999067	5.779871
C	5.277680	1.122306	5.036253
C	5.184673	0.618232	3.730237
O	8.748372	0.123181	5.808310
O	6.613620	1.464631	6.965671
H	-0.820809	-0.026077	-0.583447
H	-0.929232	-0.006108	1.931852
H	1.596847	-0.034546	2.864759
H	5.473470	-0.075989	-2.072556
H	3.167201	0.067596	-3.854048
H	1.099436	-0.129061	-2.635173
H	3.328393	1.240245	2.940366
H	6.413050	-0.825738	0.993116
H	8.346633	-0.682183	3.329228
H	4.430020	1.620941	5.507961

2-5(b)+Zn^{2+}

N	0.000000	0.000000	0.000000
C	0.000000	0.000000	1.378565
C	1.295627	0.000000	1.833354
C	2.157280	0.002473	0.677991
C	3.574154	-0.012954	0.497321
C	4.041671	-0.085694	-0.821886
C	3.153722	-0.114056	-1.927885
C	1.778384	-0.068298	-1.771709
C	1.301166	-0.010763	-0.455649
O	5.390869	-0.109522	-1.078783
O	3.787738	-0.182491	-3.150098
N	4.126679	0.716884	2.807939
C	4.498015	0.070951	1.625916
C	5.781089	-0.423279	1.798331
C	6.225343	-0.061274	3.110997
C	7.411048	-0.280882	3.850509
C	7.538099	0.231623	5.136062
C	6.445087	0.999458	5.725620
C	5.271033	1.210802	5.003621
C	5.172136	0.675373	3.711216
O	8.663560	0.034670	5.865386
O	6.588352	1.492594	6.973320
H	-0.821296	-0.019336	-0.583541
H	-0.929933	-0.005307	1.927856
H	1.590657	-0.033359	2.870797
H	5.494083	-0.074248	-2.043084
H	3.158463	-0.015658	-3.864208
H	1.111291	-0.092661	-2.627680
H	3.327504	1.325313	2.885791
H	6.324257	-0.992810	1.059944
H	8.237457	-0.847330	3.428746
H	4.461673	1.778659	5.453433
Zn	8.302179	0.911267	7.482735

2-5(a)

N	0.000000	0.000000	0.000000
C	0.000000	0.000000	1.381665
C	1.298194	0.000000	1.828289
C	2.159730	-0.000168	0.673628
C	3.588142	-0.021613	0.507372
C	4.030904	-0.119021	-0.832559
C	3.145117	-0.155618	-1.928310
C	1.764846	-0.093831	-1.780759
C	1.301537	-0.016220	-0.458270
O	5.392009	-0.144990	-1.110849
O	3.772107	-0.253697	-3.159589
N	5.838224	-0.327550	1.554411
C	4.492984	0.063952	1.627755
C	4.290966	0.519354	2.944437
C	5.508091	0.412953	3.667105
C	5.923018	0.726534	4.995309
C	7.241883	0.512460	5.452494
C	8.248246	-0.080850	4.463262
C	7.799778	-0.373450	3.143700
C	6.469963	-0.126435	2.763282
O	7.633470	0.789640	6.647903
O	9.453225	-0.296084	4.839873
H	-0.820510	-0.024448	-0.583952
H	-0.929491	-0.003705	1.931971
H	1.600521	-0.031654	2.862627
H	5.478546	-0.151555	-2.076573
H	3.148833	-0.026142	-3.861701
H	1.097041	-0.132043	-2.635061
H	6.277979	-0.611461	0.694324
H	3.367067	0.936888	3.319053
H	5.206255	1.144474	5.705543
H	8.518616	-0.787430	2.435390

2-5(a)+Zn^{2+}

N	0.000000	0.000000	0.000000
C	0.000000	0.000000	1.378099
C	1.295683	0.000000	1.831968
C	2.158833	0.002756	0.679306
C	3.578272	-0.006515	0.497139
C	4.030281	-0.077981	-0.830348
C	3.142225	-0.107487	-1.934306
C	1.768750	-0.064861	-1.773520
C	1.300812	-0.011246	-0.454142
O	5.382489	-0.103731	-1.108399
O	3.775775	-0.170298	-3.155894
N	5.839703	-0.308470	1.524495
C	4.499521	0.061290	1.630674
C	4.281845	0.466683	2.943995
C	5.513158	0.343577	3.659044
C	5.910146	0.584092	4.995795
C	7.225932	0.363998	5.382512
C	8.208795	-0.089607	4.401947
C	7.818429	-0.346204	3.088490
C	6.476935	-0.134017	2.732813
O	7.627077	0.561120	6.660488
O	9.488864	-0.254323	4.790468
H	-0.820429	-0.012654	-0.584948
H	-0.929657	-0.002596	1.927937
H	1.586603	-0.034249	2.868820
H	5.479494	-0.190016	-2.069584
H	3.126415	-0.209090	-3.870303
H	1.097443	-0.085471	-2.626006
H	6.272303	-0.555736	0.648378
H	3.356159	0.852787	3.339849
H	5.197409	0.934373	5.738169
H	8.554189	-0.696026	2.369981
Zn	9.463895	0.158976	6.630528

2-8(b)

N	0.000000	0.000000	0.000000
C	0.000000	0.000000	1.378749
C	1.297342	0.000000	1.831725
C	2.151570	-0.021692	0.675489
C	3.551051	-0.089162	0.500369
C	4.025472	-0.164623	-0.800607
C	3.172640	-0.151870	-1.928375
C	1.769894	-0.067101	-1.809079
C	1.305549	-0.020547	-0.462839
O	5.373777	-0.255394	-1.119604
O	3.746972	-0.201188	-3.181623
N	-0.434088	0.517723	-2.815518
C	0.877703	-0.015804	-2.943592
C	0.993299	-0.503521	-4.244529
C	-0.211047	-0.236736	-4.959323
C	-0.669951	-0.492752	-6.284690
C	-1.933629	-0.059953	-6.747509
C	-2.812447	0.719428	-5.772605
C	-2.326795	0.930069	-4.448296
C	-1.068150	0.450378	-4.062256
O	-2.375649	-0.282509	-7.936641
O	-3.953166	1.152339	-6.159710
H	-0.805909	-0.105780	-0.597590
H	-0.931566	0.005392	1.926114
H	1.610768	0.010410	2.866609
H	4.234364	-0.109757	1.344208
H	5.902884	0.048336	-0.370515
H	4.706088	-0.150868	-3.041310
H	-0.583933	1.325264	-2.223871
H	1.860419	-1.034603	-4.613564
H	-0.033432	-1.037245	-6.985254
H	-2.962705	1.470297	-3.745940

2-8(b)+Zn^{2+}

N	0.000000	0.000000	0.000000
C	0.000000	0.000000	1.381276
C	1.294184	0.000000	1.837182
C	2.148335	-0.021191	0.681975
C	3.545461	-0.075665	0.504937
C	4.036092	-0.139432	-0.786810
C	3.181997	-0.131081	-1.920647
C	1.788399	-0.058951	-1.790708
C	1.304267	-0.021270	-0.458750
O	5.377934	-0.213277	-1.107087
O	3.751286	-0.178867	-3.166426
N	-0.344012	0.644479	-2.861206
C	0.895497	-0.012300	-2.944924
C	1.000097	-0.577660	-4.200084
C	-0.179157	-0.237258	-4.943326
C	-0.636392	-0.518642	-6.250505
C	-1.849959	-0.002463	-6.691162
C	-2.654580	0.827505	-5.800532
C	-2.210619	1.095241	-4.505033
C	-0.985027	0.556866	-4.091738
O	-2.308135	-0.251587	-7.940525
O	-3.827554	1.321070	-6.246198
H	-0.813695	-0.109230	-0.585240
H	-0.932822	0.005703	1.925947
H	1.606309	0.010497	2.871926
H	4.222917	-0.085949	1.353970
H	5.916436	-0.024213	-0.327450
H	4.712887	-0.147294	-3.036797
H	-0.503716	1.409413	-2.221049
H	1.827747	-1.186177	-4.532491
H	-0.050177	-1.131826	-6.929971
H	-2.819844	1.708300	-3.847226
Zn	-3.945770	0.663864	-8.004324

2-8(a)

N	0.000000	0.000000	0.000000
C	0.000000	0.000000	1.377772
C	1.297245	0.000000	1.826831
C	2.145690	0.000000	0.668510
C	3.548722	0.000000	0.513614
C	4.033365	0.000000	-0.783105
C	3.187751	0.000000	-1.914077
C	1.772814	0.000000	-1.825652
C	1.301519	0.000000	-0.473720
O	5.382795	0.000000	-1.100829
O	3.804919	0.000000	-3.157627
N	1.391369	0.000000	-4.289071
C	0.897244	0.000000	-2.974962
C	-0.509206	0.000000	-3.101177
C	-0.860456	0.000000	-4.475277
C	-2.087924	0.000000	-5.201431
C	-2.132879	0.000000	-6.613076
C	-0.801215	0.000000	-7.368798
C	0.402181	0.000000	-6.608307
C	0.363680	0.000000	-5.203904
O	-3.225527	0.000000	-7.292634
O	-0.807023	0.000000	-8.648571
H	-0.827431	0.000000	-0.568418
H	-0.932153	0.000000	1.924007
H	1.617005	0.000000	2.859684
H	4.220852	0.000000	1.366483
H	5.909168	0.000000	-0.291245
H	4.761107	0.000000	-2.997719
H	2.376204	0.000000	-4.498566
H	-1.237505	0.000000	-2.302076
H	-3.038985	0.000000	-4.664660
H	1.352697	0.000000	-7.142621

2-8(a)+Zn^{2+}

N	0.000000	0.000000	0.000000
C	0.000000	0.000000	1.379679
C	1.293345	0.000000	1.832349
C	2.140977	0.000000	0.674630
C	3.540026	0.000000	0.520866
C	4.042308	0.000000	-0.764569
C	3.197411	0.000000	-1.902722
C	1.792420	0.000000	-1.809230
C	1.298975	0.000000	-0.471906
O	5.386370	0.000000	-1.078702
O	3.815762	0.000000	-3.133889
N	1.429814	0.000000	-4.282113
C	0.918719	0.000000	-2.983627
C	-0.470986	0.000000	-3.094732
C	-0.813510	0.000000	-4.479843
C	-2.034788	0.000000	-5.192156
C	-2.029216	0.000000	-6.580146
C	-0.760615	0.000000	-7.307622
C	0.444773	0.000000	-6.606633
C	0.406094	0.000000	-5.202161
O	-3.184632	0.000000	-7.283922
O	-0.782156	0.000000	-8.656367
H	-0.833237	0.000000	-0.559562
H	-0.933985	0.000000	1.922399
H	1.612205	0.000000	2.864907
H	4.204812	0.000000	1.379603
H	5.918043	0.000000	-0.272162
H	4.773528	0.000000	-2.977976
H	2.418107	0.000000	-4.480723
H	-1.200731	0.000000	-2.301557
H	-2.988940	0.000000	-4.671590
H	1.382840	0.000000	-7.154257
Zn	-2.618808	0.000000	-9.077557

5-5

N	0.000000	0.000000	0.000000
C	0.000000	0.000000	1.385266
C	1.295161	0.000000	1.836846
C	2.158450	0.003475	0.683453
C	3.578347	-0.015796	0.550175
C	4.056576	-0.019982	-0.768208
C	3.184767	-0.042390	-1.886973
C	1.802023	-0.036441	-1.762975
C	1.306041	0.000485	-0.452735
O	5.411509	0.052976	-1.023965
O	3.829746	-0.061024	-3.115689
N	4.902671	2.184566	4.574142
C	3.937579	2.939370	3.917874
C	3.587699	2.269021	2.761137
C	4.356023	1.053615	2.694395
C	4.467759	-0.014658	1.733332
C	5.365917	-1.104789	1.950269
C	6.238096	-1.089911	3.202255
C	6.101349	-0.003092	4.099828
C	5.175853	1.033720	3.841610
O	5.455572	-2.116777	1.162364
O	7.063673	-2.058581	3.401284
H	-0.820777	-0.013610	-0.583661
H	-0.933204	0.003672	1.930252
H	1.617644	0.024010	2.866406
H	5.498657	0.148642	-1.985064
H	3.209137	0.193960	-3.810851
H	1.154829	-0.061806	-2.634794
H	5.357756	2.444308	5.433833
H	3.581512	3.870290	4.335946
H	2.845891	2.596273	2.044027
H	6.721674	0.012194	4.997989

55+Zn^{2+}

N	0.000000	0.000000	0.000000
C	0.000000	0.000000	1.383256
C	1.294429	0.000000	1.839274
C	2.152214	0.000791	0.684982
C	3.561550	-0.009168	0.529523
C	4.055299	-0.012511	-0.773697
C	3.185327	-0.017237	-1.897155
C	1.805379	-0.011879	-1.762073
C	1.303559	-0.000143	-0.453628
O	5.412037	0.012778	-0.998828
O	3.837595	-0.023846	-3.113506
N	5.159468	2.429095	4.301217
C	4.188411	3.149129	3.627452
C	3.747972	2.415488	2.552205
C	4.473755	1.172352	2.554951
C	4.466285	0.030591	1.707992
C	5.311271	-1.041713	2.010598
C	6.219058	-0.963538	3.149674
C	6.231359	0.164095	3.966915
C	5.352844	1.215077	3.664396
O	5.295344	-2.176328	1.270713
O	7.045814	-2.002913	3.403931
H	-0.822144	-0.005629	-0.582473
H	-0.933033	0.000891	1.927908
H	1.612315	0.010882	2.871075
H	5.537084	0.068671	-1.958777
H	3.205053	0.108748	-3.831704
H	1.155807	-0.016481	-2.632374
H	5.646030	2.738893	5.126940
H	3.890641	4.127038	3.977465
H	2.985755	2.711768	1.846100
H	6.909636	0.204193	4.814321
Zn	6.556936	-3.277053	2.113225

5-8

N	0.000000	0.000000	0.000000
C	0.000000	0.000000	1.379461
C	1.296906	0.000000	1.836632
C	2.151612	0.036307	0.679478
C	3.548074	0.027705	0.481459
C	4.007301	0.075132	-0.824790
C	3.136330	0.119798	-1.946921
C	1.741721	0.082365	-1.806626
C	1.304375	0.051796	-0.459591
O	5.358591	0.090409	-1.156522
O	3.710415	0.255800	-3.191631
N	-2.099328	2.099906	-3.791450
C	-1.692572	2.846429	-2.693739
C	-0.568597	2.241964	-2.162026
C	-0.269371	1.073021	-2.951646
C	0.775228	0.077234	-2.925295
C	0.800058	-0.983803	-3.884092
C	-0.262203	-0.995354	-4.981324
C	-1.250976	0.017843	-4.966805
C	-1.246269	1.016691	-3.967855
O	1.649949	-1.945592	-3.854146
O	-0.228917	-1.922027	-5.872760
H	-0.792150	0.187196	-0.597598
H	-0.931059	-0.010417	1.928435
H	1.606476	-0.029867	2.872670
H	4.246875	-0.012259	1.313064
H	5.872561	0.381752	-0.392196
H	4.662230	0.362025	-3.035628
H	-2.891893	2.311317	-4.375470
H	-2.236428	3.728710	-2.388181
H	-0.013800	2.595864	-1.302077
H	-2.016895	0.007610	-5.744053

5-8+Zn^{2+}

N	0.000000	0.000000	0.000000
C	0.000000	0.000000	1.380737
C	1.295651	0.000000	1.837949
C	2.150272	0.019456	0.682008
C	3.546249	0.020642	0.485950
C	4.022317	0.047569	-0.813274
C	3.153202	0.068115	-1.938819
C	1.766180	0.043726	-1.788670
C	1.302458	0.027447	-0.456804
O	5.365588	0.057268	-1.147013
O	3.708542	0.140414	-3.190606
N	-1.799929	2.298354	-4.007881
C	-1.402700	3.001431	-2.885648
C	-0.370788	2.329563	-2.274429
C	-0.113227	1.142750	-3.049777
C	0.818482	0.072588	-2.930715
C	0.786744	-0.960273	-3.874824
C	-0.154262	-0.907838	-4.989952
C	-1.053245	0.148823	-5.106264
C	-1.027703	1.156466	-4.130826
O	1.601885	-2.033305	-3.766801
O	-0.147787	-1.905181	-5.901442
H	-0.808209	0.108228	-0.592910
H	-0.931768	-0.004866	1.927563
H	1.605203	-0.015874	2.873672
H	4.237164	0.004476	1.324681
H	5.897006	0.265366	-0.367480
H	4.667533	0.219308	-3.065588
H	-2.541187	2.570746	-4.633238
H	-1.893060	3.925387	-2.615359
H	0.146462	2.638650	-1.377455
H	-1.752700	0.168092	-5.936889
Zn	1.122825	-3.110148	-5.224974
